# ﻿Micromorphological leaf epidermal traits as potential taxonomic markers for infrageneric classification of *Oxytropis* (Fabaceae)

**DOI:** 10.3897/phytokeys.201.85154

**Published:** 2022-06-21

**Authors:** Xiang Zhao, Qinzheng Hou, Meina Du, Hui Zhang, Lingyun Jia, Zhihua Zhang, Zongqi Ma, Kun Sun

**Affiliations:** 1 College of Life Sciences, Northwest Normal University, Lanzhou 730070, Gansu, China Northwest Normal University Lanzhou China

**Keywords:** China, cluster analysis, leaf epidermis, LM, *Oxytropis*, SEM, taxonomy

## Abstract

The characteristics of the leaf epidermis have proven to be useful criteria to support taxonomic studies within Fabaceae. However, there are few systematic studies on the taxonomic significance of leaf epidermis of *Oxytropis* DC. Here, we used light and scanning electron microscopy to investigate leaf epidermal characteristics of 18 species of genus *Oxytropis* from the Northeastern Margin of Qinghai-Tibet Plateau. Our examination showed two main types of leaf epidermal cells: polygonal and irregular, and four different patterns of anticlinal walls: straight-arched, sinuolate, undulate, and sinuate. All species studied possess anomocytic stomata. Two trichome shapes were identified: strip-like trichomes, that were present only in *O.ciliata*, and cylindrical trichomes, present in all other species. Epidermal cell shape and anticlinal wall pattern were constant within species and are useful for species delimitation within genus *Oxytropis*, when combined with other macroscopic traits. The shape of trichomes can be useful for distinguishing *O.ciliata* from the other investigated species. Stomatal type was the same within the genus and may be used to elaborate the phylogenetic relationships between genera in combination with data on stomata from other genera. Cluster analysis results were largely consistent with the classification of species and sections based on macro morphological data, indicating that foliar epidermis characteristics of *Oxytropis* can be used as markers for taxonomic identification at the infrageneric classification level. Lastly, our results support the delineation of the section Leucopodia as an independent section but do not support the merging of section Gobicola into section Baicalia.

## ﻿Introduction

Genus *Oxytropis* DC. is one of the largest groups within Fabaceae, with approximately 330 species occurring in the cold mountainous regions of Europe, Asia, and North America, and also concentrated in Central Asia (Polhill 1981; Zhu et al. 2010). The genus was established by De Candolle (1802), who distinguished it from *Astragalus* based on differences in keel-petals and legumes. In China, *Oxytropis* species are mainly distributed in the north and northwest regions (Zhang 1998; Zhu and Ohashi 2000). However, there is some debate regarding the delimitation and identification of species within this genus. In China, the genus was first reported by Peter-Stibal (1937), who recorded two subgenera, 11 sections, and 27 species. Wang and Tang (1955) recorded 27 species of *Oxytropis* and 1 variety in China. In contrast, in Flora Reipublica Popularis Sinicae (FRPS), Zhang (1998) divided *Oxytropis* into six subgenera, 22 sections, and 146 species. While Zhu and Ohashi (2000) recognized 125 species and 4 varieties, a recent study (Zhu et al. 2010) on the Flora of China (FOC) reported that the genus *Oxytropis* consisted of three subgenera and 20 sections containing 133 species. Therefore, different species delimitations in China have been proposed by taxonomists, whereby the infrageneric delimitation of *Oxytropis* remains controversial.

Leaf epidermal anatomical features, such as epidermal cell shape, epicuticular waxes (Barthlott et al. 1998; Wissemann 2000; Tomaszewski and Zieliński 2014; Tomaszewski et al. 2019), stomatal complexes (Carpenter 2005; Alvarez et al. 2009; Yang et al. 2012; Nisa et al. 2019), and trichomes (Webster et al. 1996; Hu et al. 2012; Eiji and Salmaki 2016; Mannethody and Purayidathkandy 2018; Ashfaq et al. 2019), are all useful diagnostic and taxonomic characteristics. The taxonomic relevance of the foliar epidermal characteristics of Fabaceae is well documented (Zou et al. 2008; Alege and Shaibu 2015; Silva et al. 2018; Shaheen et al. 2020). Zou et al. (2008) found that epidermal characteristics can be used to distinguish genus *Bauhinia* from *Cercis*. Similarly, Chukwuma et al. (2014) described the presence of glandular trichomes and found that they could be used as a distinguishing feature between genera *Centrosema* and *Clitoria*. Consistently, in genus *Lotus*, epidermal micromorphological features are useful and informative for distinguishing between sections *Simpeteria* and *Microlotus* (Stenglein et al. 2003).

Leaf epidermal features are also valuable for classification at the species level in Fabaceae. For example, Silva et al. (2018) found that leaflet anatomy is taxonomically useful at both genus and species levels in the Dipterygeae clade. Similarly, Rashid et al. (2019) concluded that the combination of leaf epidermal characteristics and other traits has potential for taxonomic resolution at the species level in the tribe *Trifolieae*. Additionally, in *Crotalaria*, leaf characteristics, such as texture, venation pattern, and epidermis, have shown potential for aiding the circumscription of some species (Devecchi et al. 2014).

**Figure 1.**
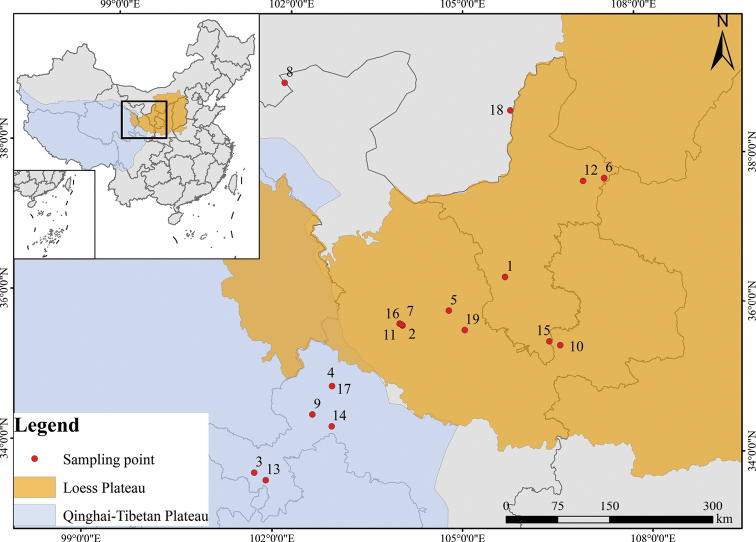
Map of study area. Numbers represent sample codes, as shown in Table 1.

Previous studies have investigated different aspects of *Oxytropis*, including cytology (Ledingham 1957, 1960; Ledingham and Rever 1963; Ranjbar et al. 2010; Liu et al. 2011; Martin et al. 2015), molecular phylogeny (Jorgensen et al. 2003; Archambault and Strömvik 2012; Dizkirici et al. 2016), pollen (Zhu and Ohashi 2000; Wang 2005; Ceter et al. 2013), and seed micromorphology (Solum and Lockerman 1991; Bojňanský and Fargašová 2007; Meyers et al. 2013; Erkul et al. 2015). On the other hand, relatively few studies on leaf epidermal anatomy have been reported in *Oxytropis* species (Karaman et al. 2009; Lu 2011), which described leaf epidermal traits by light or scanning electron microscopy but lacked systematic analysis. In addition, the Northeastern Margin of the Qinghai-Tibet Plateau is located in the transition zone between the Qinghai-Tibet Plateau and the Loess Plateau, the two main distribution areas of *Oxytropis* in China (Fig. 1). This region includes the northeastern part of the Qinghai-Tibet Plateau and the western part of the Loess Plateau (Tian et al. 2021) (Fig. 1). It is one of the regions with rich diversity of *Oxytropis* (Zhang 1998; Zhu et al. 2010). However, little research has been conducted on the epidermal traits of *Oxytropis* in this region. Thus, we provide the first systematic comparison and microscopic investigation of 18 species of *Oxytropis* from this region using light and scanning electron microscopy to elucidate the taxonomic significance of leaf micromorphology and test the recent taxonomic treatment (Zhu et al. 2010).

## ﻿Materials and methods

All leaf samples were obtained from specimens deposited at the herbarium of the Northwest Normal University. The materials investigated are listed in Table 1, and the infrageneric classification by Zhu et al. (2010) was adopted (Fig. 1). Those used for analysis by light microscopy were soaked in water at 37 °C for 12–18 h; removed and placed under a dissecting microscope to separate the epidermal tissue from the leaf body, followed by maceration in 1% safranine solution. To check the consistency of the epidermal structure under a light microscope (DM6 B Leica, Leica Microsistemas S.L.U., Barcelona, Spain), at least 20 slides were prepared from different parts of a single leaf, and from different leaves of each species. The number and size of the stomata on each slide were counted. Materials for observation by scanning electron microscopy (SEM) were mounted directly on the stubs without treatment. After gold sputtering, the specimens were examined and imaged using a field emission (FE-SEM) Zeiss Ultra Plus instrument (Zeiss, Germany). Quantitative and qualitative traits were selected when performing cluster analysis.

**Table 1.** Source of materials.

Section	Code	Species	Locality	Coordinates	Habitat	Voucher
Section Xerobia	1	*O.ciliata*	Yueliang Mountain	36°25'41.85"N, 105°42'23.71"E	Valley	X. Zhao 1947
Section Polyadena	2	*O.muricata*	Maxian Mountain	35°47'46.48"N, 103°58'12.64"E	Sunny hillside	X. Zhao 1903
Section Falcicarpae	3	*O.falcata*	Awangcang wetland park	33°45'32.85"N, 101°41'6.58"E	Riverside	X. Zhao 1842
Section Baicalia	4	*O.ochrantha*	Dangzhou grassland	34°56'54.09"N, 102°53'8.74"E	Alpine meadow	X. Zhao 1813
	5	*O.bicolor*	Tiemu Mountain	35°58'32.21"N, 104°46'31.40"E	Sunny hillside	X. Zhao 1927
	6	*O.racemosa*	Yanchi	37°43'52.02"N, 107°23'55.77"E	Desert sandy land	X. Zhao 1946
	7	*O.myriophylla*	Maxian Mountain	35°47'46.48"N, 103°58'12.64"E	Valley	X. Zhao 1833
Section Lycotriche	8	*O.aciphylla*	Jijiquan nature reserve	38°59'43"N, 101°55'39"E	Desert sandy land	X. Zhao 1924
Section Eumorpha	9	*O.imbricata*	Taohe river	34°33'28.66"N, 102°34'53.99"E	Riverside	X. Zhao 1940
	10	*O.coerulea*	Taitong Mountain	35°30'8.94"N, 106°35'54.90"E	Border of Forest	X. Zhao 1832
Section Mesogaea	11	*O.xinglongshanica*	Xinglong Mountain	35°46'20.53"N, 104°1'2.49"E	Valley	X. Zhao 1913
	12	*O.glabra*	Rabah Lake National Nature Reserve	37°42'3.19"N, 107°2'33.46"E	Desert sandy land	X. Zhao 1950
	13	*O.kansuensis*	Azi Test Station of LZU	33°39'57.96"N, 101°52'22.44"E	Alpine meadow	X. Zhao 1819
	14	*O.melanocalyx*	Guanggai Mountain	34°24'23.35"N, 102°53'58.80"E	Alpine meadow	X. Zhao 1956
	15	*O.taochensis*	Liupan Mountain	35°33'21.81"N, 106°25'21.54"E	Border of Forest	X. Zhao 1838
	16	*O.ochrocephala*	Xinglong Mountain	35°47'5.17"N, 104°0'0.67"E	Beside farmland	X. Zhao 1828
	17	*O.ochrocephala*	Dangzhou grassland	34°56'54.11"N, 102°53'8.81"E	Alpine meadow	X. Zhao 1812
Section Oxytropis	18	*O.latibracteata*	Helan Mountain	38°39'46.59"E 105°49'20.25"N	Border of Forest	X. Zhao 1951
Section Leucopodia	19	*O.squammulosa*	Shaochagou	35°42'57.20"N, 105°2'21.20"E	Arid steppe	X. Zhao 1928

Leaf epidermal terminology was based on the classification proposed by Dilcher (1974) and Wilkinson (1980). Stomatal index (SI) was calculated using the following equation:

SI = S/E + S (1)

where, SI is the stomatal index, S is the number of stomata per unit area, and E is the number of epidermal cells per unit area. Stomatal density (SD) was expressed as the number of stomata per unit leaf area.

### ﻿Data analysis

Statistical data was processed by the Origin 2021 software (OriginLab Corporation 2021). The raw data matrix includes quantitative and qualitative characters, and qualitative traits were coded using a presence/absence (0/1) matrix (Table 2). The Euclidean distance is one of the most commonly used distance measurement methods in hierarchical clustering, which can reflect the absolute difference of individual numerical characteristics and is suitable for the analysis that needs to reflect the difference from the numerical size of the dimension (Raymond and Sylvia 1993; Farhana and Safwana 2018). The Ward error sum of squares method applies the idea of ANOVA to classification, and the obtained clustering information is more abundant and rarely affected by abnormal data (Ward 1963; Szekely and Rizzo 2005). Therefore, the Ward’s method was used for cluster analysis using the squared Euclidean distance to interpret the morpho-anatomical similarity among species in this study.

**Table 2.** Matrix of qualitative leaf epidermal characters of *Oxytropis* species.

Species	Adaxial epidermis							Abaxial epidermis						
	Shape of trichromes	Ornamentation of trichromes	Inner margin of outer stomatal rim	Ornamentation of outer stomatal rim	Waxy layer of epidermal cells	Shape of cells	Pattern of anticlinal walls	Shape of trichromes	Ornamentation of trichromes	Inner margin of outer stomatal rim	Ornamentation of outer stomatal rim	Waxy layer of epidermal cells	Shape of cells	Pattern of anticlinal walls
*O.ciliata*	0	0	0	1	1	0	0	2	2	1	1	1	1	2
*O.muricata*	1	1	1	1	1	0	0	1	1	0	0	0	0	1
*O.falcata*	1	1	0	0	2	0	1	1	1	0	0	0	0	1
*O.ochrantha*	1	0	0	1	0	0	0	1	0	1	1	1	0	3
*O.bicolor*	1	1	1	1	1	0	0	1	1	1	1	1	0	3
*O.racemosa*	1	1	0	0	2	1	2	1	1	0	0	0	0	3
*O.myriophylla*	1	0	0	0	1	1	2	1	0	0	0	2	1	2
*O.aciphylla*	1	1	0	2	2	1	2	1	1	0	0	0	1	2
*O.imbricata*	1	1	0	0	0	1	2	1	1	0	1	1	1	2
*O.coerulea*	1	1	0	3	0	0	0	2	0	0	3	0	0	1
*O.xinglongshanica*	1	1	0	2	2	1	2	1	1	0	2	2	1	2
*O.glabra*	1	1	1	0	1	1	2	1	1	0	0	0	0	3
*O.kansuensis*	1	1	0	0	0	1	2	1	1	0	0	0	0	3
*O.melanocalyx*	1	1	0	2	2	1	2	1	1	0	1	1	0	3
*O.taochensis*	1	1	0	2	2	1	2	1	1	0	0	0	0	3
*O.ochrocephala* (XLS)	1	1	0	2	2	1	2	1	1	0	1	1	0	1
*O.ochrocephala* (HZ)	1	1	0	2	2	1	2	1	1	0	1	1	0	1
*O.latibracteata*	1	1	1	1	1	0	3	1	1	0	1	1	0	1
*O.squammulosa*	2	2	0	0	0	0	3	2	2	0	0	0	0	1

Note: Shape of trichromes: strip-like 0, cylindrical 1, absent 2; Ornamentation of trichromes: striation 0, striation and granular 1, absent 2; Inner margin of outer stomatal rim: undulate 0, smooth 1; Ornamentation of outer stomatal rim: granular 0, smooth 1, scale-like 2, banded sediment 3; Waxy layer of epidermal cells: granular 0, smooth 1, scale-like 2; Shape of cells: irregular 0, polygonal 1; Pattern of anticlinal walls: sinuate 0, sinuolate 1, straight arched 2, Undulate 3.

## ﻿Results

### ﻿Epidermal cell characters

**Figure 2.**
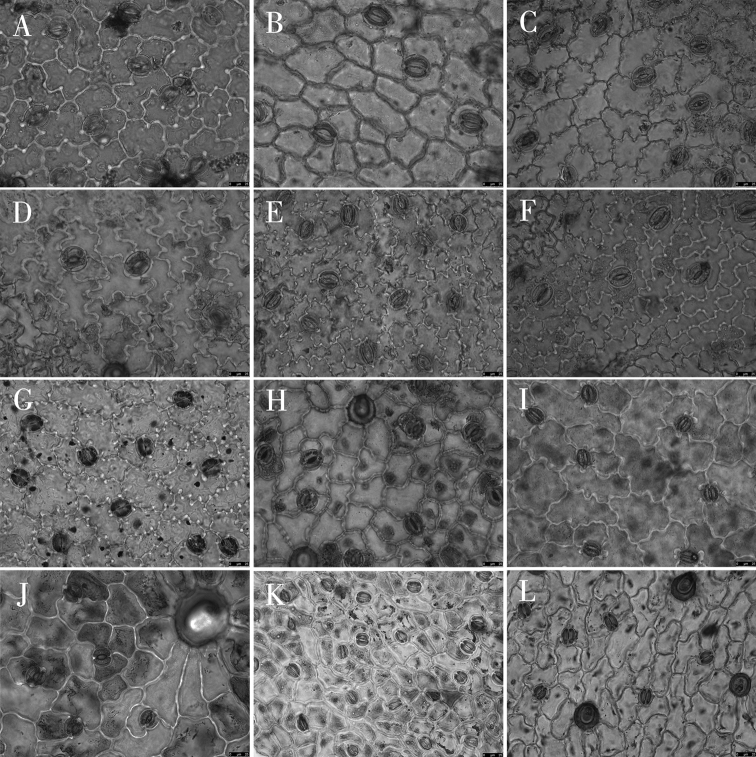
Light microscope photographs of epidermal cells in *Oxytropis* DC. **A, B** adaxial and abaxial epidermall cells of *O.ciliata***C, D** adaxial and abaxial epidermall cells of *O.muricata***E, F** adaxial and abaxial epidermall cells of *O.falcata***G, H** adaxial and abaxial epidermall cells of *O.ochrantha***I, J** adaxial and abaxial epidermall cells of *O.bicolor***K, L** adaxial and abaxial epidermall cells of *O.racemosa*.

**Figure 3.**
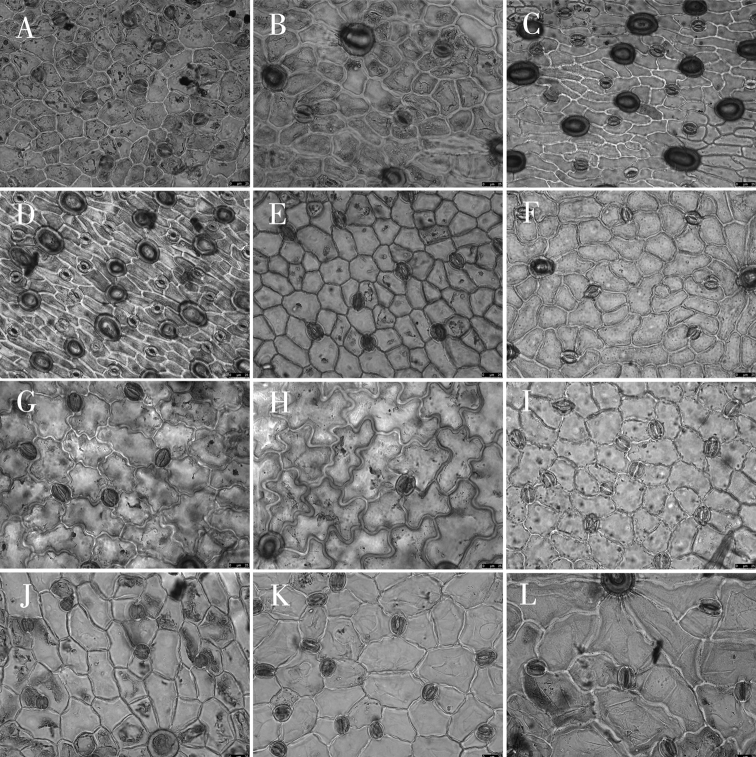
Light microscope photographs of epidermal cells in *Oxytropis* DC. **A, B** adaxial and abaxial epidermall cells of *O.myriophylla***C, D** adaxial and abaxial epidermall cells of *O.aciphylla***E, F** adaxial and abaxial epidermall cells of *O.imbricata***G, H** adaxial and abaxial epidermall cells of *O.coerulea***I, J** adaxial and abaxial epidermall cells of *O.xinglongshanica***K, L** adaxial and abaxial epidermall cells of *O.glabra*.

**Figure 4.**
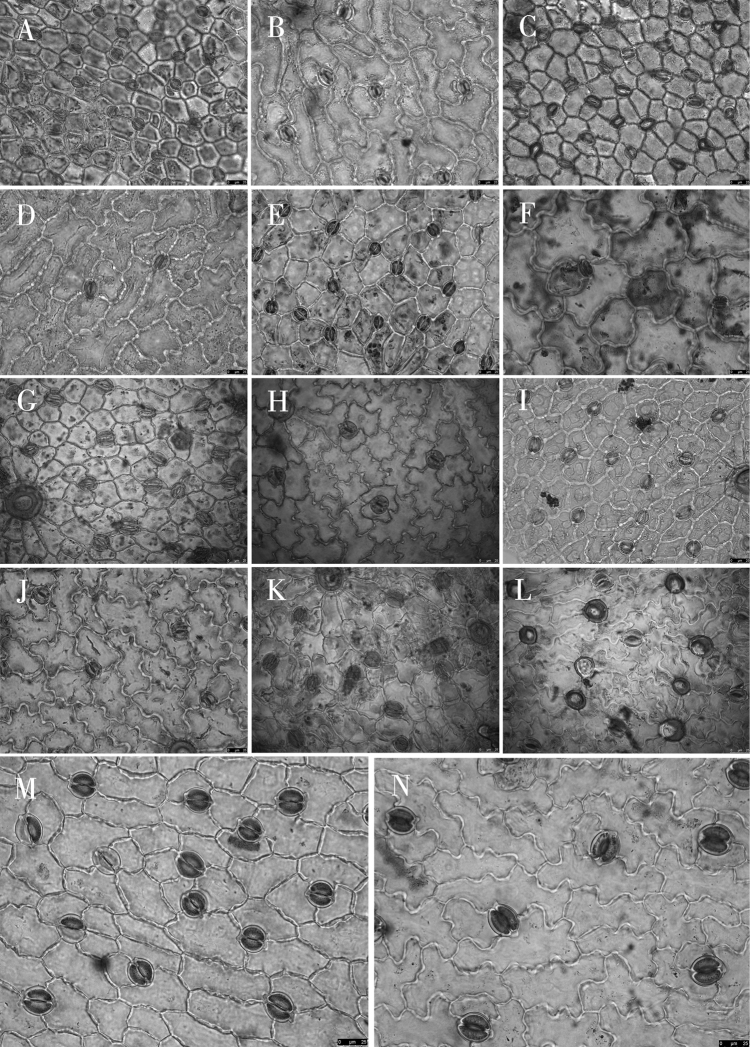
Light microscope photographs of epidermal cells in *Oxytropis* DC. **A, B** adaxial and abaxial epidermall cells of *O.kansuensis***C, D** adaxial and abaxial epidermall cells of *O.melanocalyx***E, F** adaxial and abaxial epidermall cells of *O.taochensis***G, H** adaxial and abaxial epidermall cells of *O.ochrocephala* (XLS) **I, J** adaxial and abaxial epidermall cells of *O.ochrocephala* (HZ) **K, L** adaxial and abaxial epidermall cells of *O.latibracteata***M, N** adaxial and abaxial epidermall cells of *O.squammulosa*.

Epidermal cell traits varied within a wide range. The shape varied from polygonal to irregular with straight arched, sinuolate, undulate, and sinuate wall patterns (Table 3; Figs 2–4). Polygonal cells with straight-arched walls were common in most taxa and were predominant in *O.racemosa*, *O.glabra*, *O.kansuensis*, *O.melanocalyx*, *O.taochensis*, *O.ochrocephala* (XLS), *O.ochrocephala* (HZ), *O.myriophylla*, *O.aciphylla*, *O.imbricata*, *O.xinglongshanica*, and *O.ciliata* (Figs 2–4). In turn, irregular sinuolate walls were predominant in *O.ciliata*, *O.muricata*, *O.ochrantha*, *O.bicolor*, and *O.coerulea* (Figs 2, 3), and irregular undulate walls were predominant in *O.latibracteata*, *O.squammulosa*, *O.ochrantha*, *O.bicolor*, *O.racemosa*, *O.glabra*, *O.kansuensis*, *O.melanocalyx*, and *O.taochensis* (Figs 2–4). Lastly, irregular sinuate walls were predominant in *O.falcata*, *O.muricata*, *O.coerulea*, *O.ochrocephala* (XLS), *O.ochrocephala* (HZ), *O.latibracteata*, and *O.squammulosa* (Figs 2–4).

**Table 3.** Characteristics of the leaf epidermis of *Oxytropis* under light microscopy (surface view).

Species	Adaxial epidermis							Abaxial epidermis						
	Shape of cells	Pattern of anticlinal walls	Type of stomata	Mean stomatal density /(mm^2^)	Mean stomatal index /%	Mean stomatal size /mm^2^	Shape of cells	Pattern of anticlinal walls	Type of stomata	Mean stomatal density /(mm^2^)	Mean stomatal index /%	Mean stomatal size /mm^2^	Adaxial and abaxial SD ratio	Adaxial and abaxial SI ratio
*O.ciliata*	Irregular	Sinuolate	Anomocytic	131.77	0.21	639.84 (27.88×22.95)	Polygonal	Straight arched	Anomocytic	63.54	0.11	748.35 (30.2×24.78)	2.07	1.90
*O.muricata*	Irregular	Sinuolate	Anomocytic	129.92	0.17	647.79 (29.01×22.33)	Irregular	Sinuate	Anomocytic	66.14	0.11	810.79 (31.61×25.65)	1.96	1.54
*O.falcata*	Irregular	Sinuate	Anomocytic	170.87	0.17	571.99 (26.42×21.65)	Irregular	Sinuate	Anomocytic	81.99	0.1	669.96 (28.94×23.15)	2.08	1.7
*O.ochrantha*	Irregular	Sinuolate	Anomocytic	156.4	0.19	511.08 (24.69×20.7)	Irregular	Undulate	Anomocytic	95.08	0.1	541.54 (25.69×21.08)	1.64	1.9
*O.bicolor*	Irregular	Sinuolate	Anomocytic	110.24	0.23	448.58 (24.58×18.25)	Irregular	Undulate	Anomocytic	77.17	0.16	497.51 (23.59×21.09)	1.42	1.43
*O.racemosa*	Polygonal	Straight arched	Anomocytic	292.82	0.18	312.63 (19.18×16.3)	Irregular	Undulate	Anomocytic	97.15	0.09	357.39 (21.7×16.47)	3.01	2
*O.myriophylla*	Polygonal	Straight arched	Anomocytic	250.79	0.15	410.40 (21.83×18.8)	Polygonal	Straight arched	Anomocytic	33.07	0.03	423.75 (23.82×17.79)	7.58	5
*O.aciphylla*	Polygonal	Straight arched	Anomocytic	369.29	0.16	253.77 (16.84×15.07)	Polygonal	Straight arched	Anomocytic	234.94	0.11	257.21 (17.45×14.74)	1.57	1.45
*O.imbricata*	Polygonal	Straight arched	Anomocytic	139.17	0.14	409.05 (22.7×18.02)	Polygonal	Straight arched	Anomocytic	81.3	0.11	372.01 (21.96×16.94)	1.71	1.27
*O.coerulea*	Irregular	Sinuolate	Anomocytic	152.95	0.21	526.83 (25.28×20.84)	Irregular	Sinuate	Anomocytic	0.69	0.0031	514.8 (26.4×19.5)	221.66	67.74
*O.xinglongshanica*	Polygonal	Straight arched	Anomocytic	209.45	0.19	403.65 (21.89×18.44)	Polygonal	Straight arched	Anomocytic	67.18	0.09	389.68 (21.03×18.53)	3.11	2.11
*O.glabra*	Polygonal	Straight arched	Anomocytic	173.62	0.25	442.83 (24.08×18.39)	Irregular	Undulate	Anomocytic	92.32	0.21	517.17 (26.95×19.19)	1.88	1.19
*O.kansuensis*	Polygonal	Straight arched	Anomocytic	412.7	0.22	251.78 (17.87×14.09)	Irregular	Undulate	Anomocytic	63.39	0.13	389.15 (22.25×17.49)	6.51	1.69
*O.melanocalyx*	Polygonal	Straight arched	Anomocytic	383.53	0.26	376.79 (21.73×17.34)	Irregular	Undulate	Anomocytic	39.96	0.09	368.32 (22.68×16.24)	9.59	2.88
*O.taochensis*	Polygonal	Straight arched	Anomocytic	202.56	0.21	418.08 (23.37×17.89)	Irregular	Undulate	Anomocytic	36.99	0.12	373.49 (21.88×17.07)	5.47	1.75
*O.ochrocephala* (XLS)	Polygonal	Straight arched	Anomocytic	265.95	0.2	388.29 (21.56×18.01)	Irregular	Sinuate	Anomocytic	58.25	0.11	418.50 (22.72×18.42)	4.56	1.81
*O.ochrocephala* (HZ)	Polygonal	Straight arched	Anomocytic	289.37	0.21	419.94 (22.91×18.33)	Irregular	Sinuate	Anomocytic	57.87	0.11	448.21 (23.64×18.96)	5.0003	1.90
*O.latibracteata*	Irregular	Undulate	Anomocytic	147.64	0.16	485.93 (24.53×19.81)	Irregular	Sinuate	Anomocytic	93.21	0.1	544.02 (26.03×20.9)	1.58	1.6
*O.squammulosa*	Irregular	Undulate	Anomocytic	226.67	0.22	465.37 (22.58×20.61)	Irregular	Sinuate	Anomocytic	99.9	0.15	542.38 (25.84×20.99)	2.26	1.46

Note: XLS (Xinglongshan population); HZ (Hezuo population)

**Figure 5.**
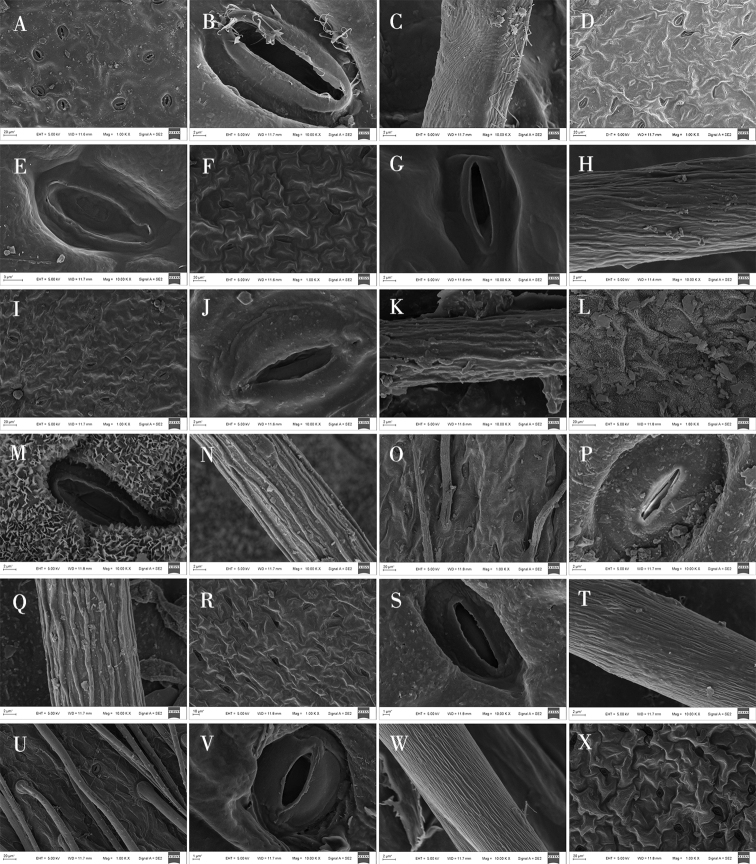
Scanning electron microscope photographs of epidermal cells in *Oxytropis* DC. **A–C** adaxial epidermall cells of *O.ciliata***D, E** abaxial epidermall cells of *O.ciliata***F–H** adaxial epidermall cells of *O.muricata***I–K** abaxial epidermall cells of *O.muricata***L–N** adaxial epidermall cells of *O.falcata***O–Q** abaxial epidermall cells of *O.falcata***R–T** adaxial epidermall cells of *O.ochrantha*. **U–W** abaxial epidermall cells of *O.ochrantha***X** adaxial epidermall cells of *O.bicolor*.

**Figure 6.**
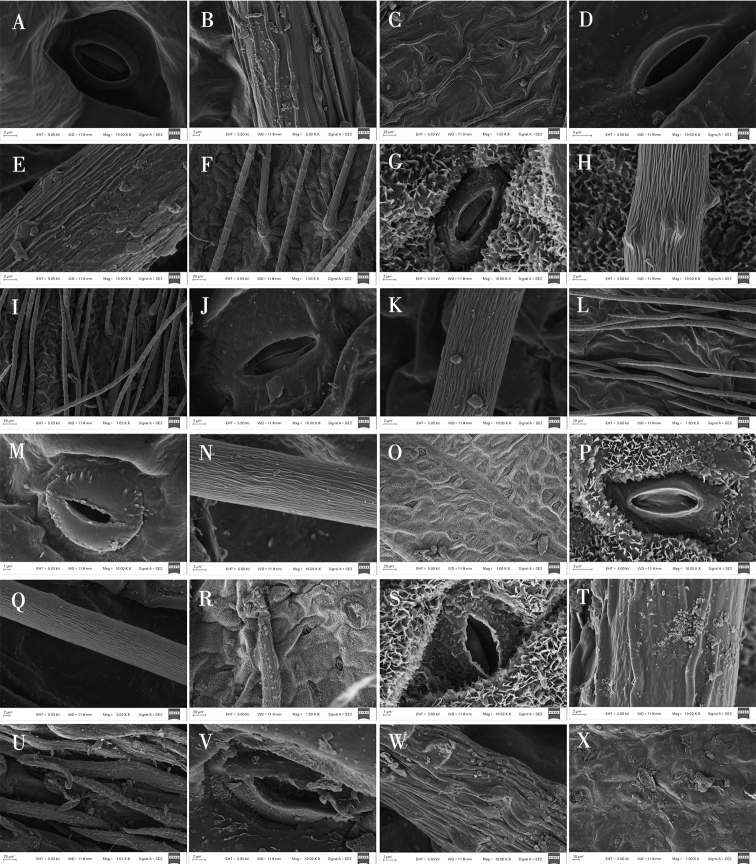
Scanning electron microscope photographs of epidermal cells in *Oxytropis* DC. **A, B** adaxial epidermall cells of *O.bicolor***C–E** abaxial epidermall cells of *O.bicolor***F–H** adaxial epidermall cells of *O.racemosa***I–K** abaxial epidermall cells of *O.racemosa***L–N** adaxial epidermall cells of *O.myriophylla***O–Q** abaxial epidermall cells of *O.myriophylla***R–T** adaxial epidermall cells of *O.aciphylla***U–W** abaxial epidermall cells of *O.aciphylla***X** adaxial epidermall cells of *O.imbricata*.

**Figure 7.**
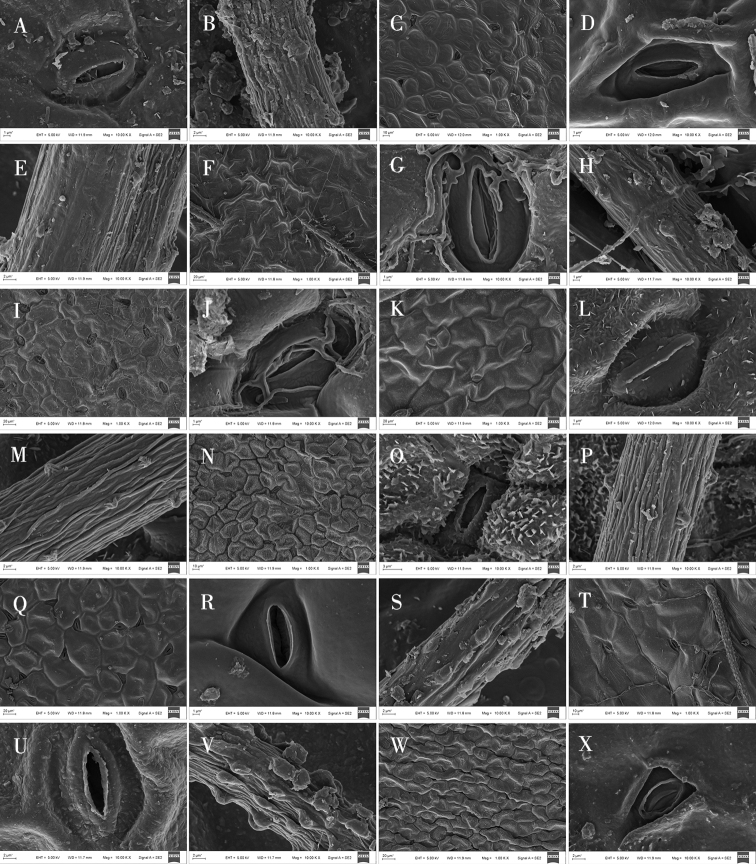
Scanning electron microscope photographs of epidermal cells in *Oxytropis* DC. **A, B** adaxial epidermall cells of *O.imbricata***C–E** abaxial epidermall cells of *O.imbricata***F–H** adaxial epidermall cells of *O.coerulea***I, J** abaxial epidermall cells of *O.coerulea***K–M** adaxial epidermall cells of *O.xinglongshanica***N–P** abaxial epidermall cells of *O.xinglongshanica***Q–S** adaxial epidermall cells of *O.glabra***T–V** abaxial epidermall cells of *O.glabra***W–X** adaxial epidermall cells of *O.kansuensis*.

**Figure 8.**
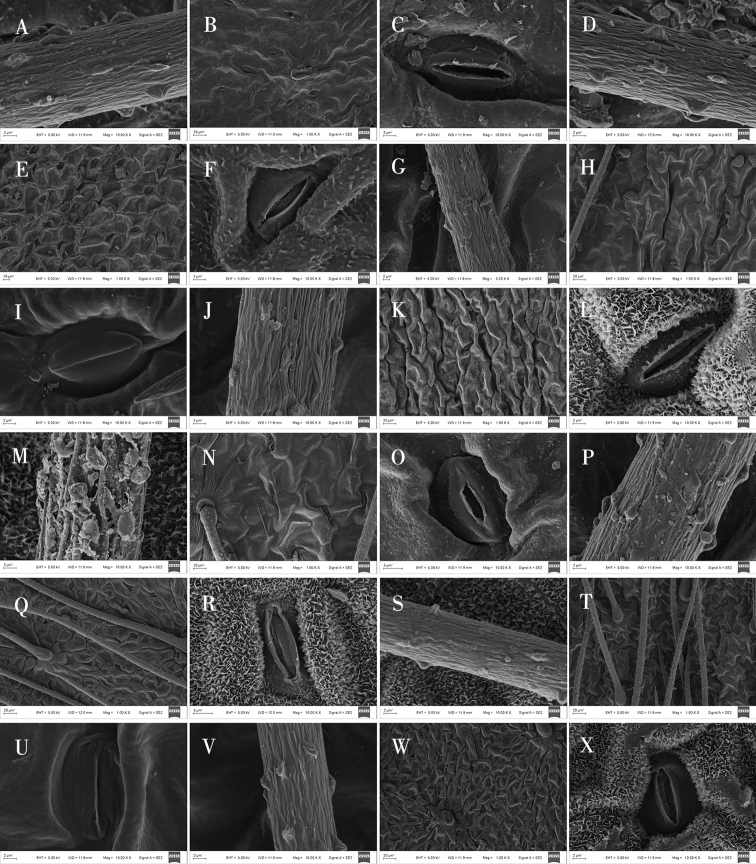
Scanning electron microscope photographs of epidermal cells in *Oxytropis* DC. **A** adaxial epidermall cells of *O.kansuensis***B–D** abaxial epidermall cells of *O.kansuensis***E–G** adaxial epidermall cells of *O.melanocalyx***H–J** abaxial epidermall cells of *O.melanocalyx***K–M** adaxial epidermall cells of *O.taochensis***N–P** abaxial epidermall cells of *O.taochensis***Q–S** adaxial epidermall cells of *O.ochrocephala* (HZ) **T–V** abaxial epidermall cells of *O.ochrocephala* (HZ) **W–X** adaxial epidermall cells of *O.ochrocephala* (XLS).

**Figure 9.**
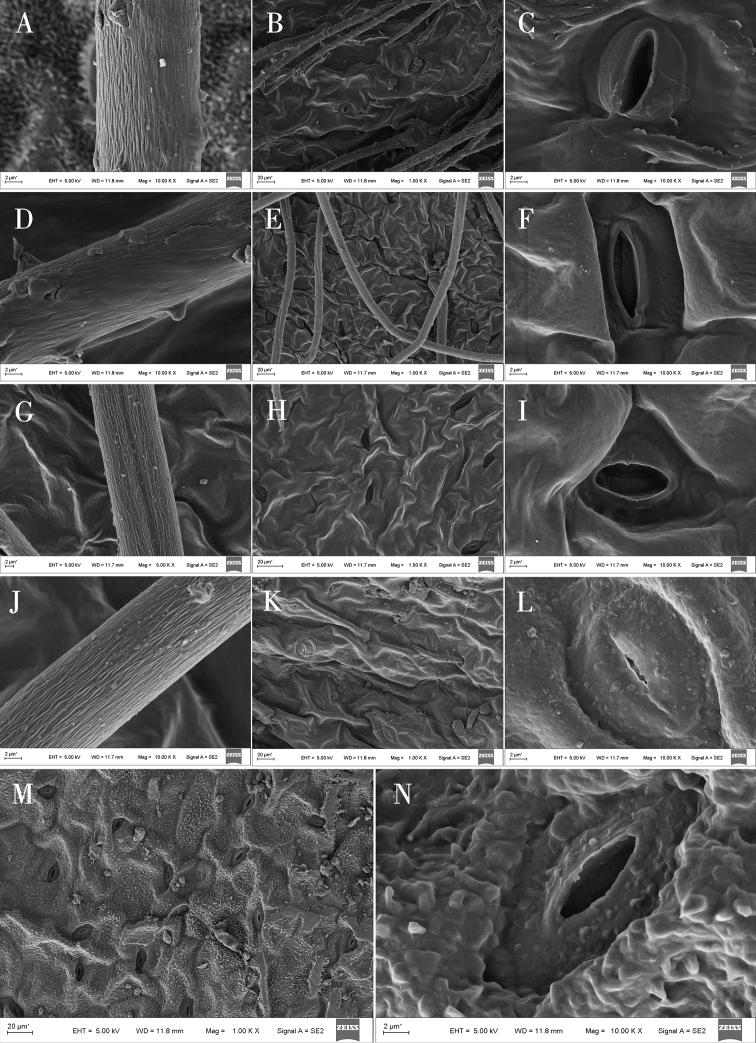
Scanning electron microscope photographs of epidermal cells in *Oxytropis* DC. **A** adaxial epidermall cells of *O.ochrocephala* (XLS) **B–D** abaxial epidermall cells of *O.ochrocephala* (XLS) **E–G** adaxial epidermall cells of *O.latibracteata***H–J** abaxial epidermall cells of *O.latibracteata***K–L** adaxial epidermall cells of *O.squammulosa***M–N** abaxial epidermall cells of *O.squammulosa*.

In addition, SEM analysis showed that, based on the shape, the waxy layer on epidermal cells could be separated into three groups (Table 4): a smooth waxy layer was found in epidermal cells of *O.ciliata*, *O.muricata*, *O.bicolor*, *O.myriophylla*, *O.glabra*, *O.ochrantha*, *O.ochrocephala* (XLS), *O.ochrocephala* (HZ), *O.imbricata*, *O.melanocalyx*, and *O.latibracteata* (Figs 5–9); a granular waxy layer was observed in those of *O.ochrantha*, *O.imbricata*, *O.kansuensis*, *O.racemosa*, *O.muricata*, *O.falcata*, *O.aciphylla*, *O.glabra*, *O.kansuensis*, *O.ochrocephala* (XLS), *O.ochrocephala* (HZ), and *O.squammulosa* (Figs 5–9), and finally, a scale-like waxy layer was observed in epidermal cells of *O.falcata*, *O.racemosa*, *O.aciphylla*, *O.xinglongshanica*, *O.melanocalyx*, *O.taochensis*, *O.ochrocephala* (XLS), and *O.ochrocephala* (HZ) (Figs 5–9).

### ﻿Stomatal characters on the epidermis

With respect to stomata, all species of *Oxytropis* studied here were anomocytic, and stomatal index (SI) and stomatal density (SD) of the adaxial epidermis were greater than those of the abaxial epidermis (Table 3). In most of the examined species, remarkable variation was observed in stomatal size and number. Specifically, SD was lowest in *O.coerulea* (Table 3; Fig. 3) and largest in *O.melanocalyx* (Table 3; Fig. 4). Meanwhile, SI was highest (0.26) in *O.melanocalyx*, and lowest (0.003) on the abaxial surface of *O.coerulea* (Table 3; Figs 3, 4). Stomatal size was largest in *O.muricata* and smallest in *O.aciphylla* (Table 3; Figs 2, 3). As per SEM observation, the inner margin of the outer stomatal rim was either undulate or smooth (Table 4). Five species, including *O.ciliata*, *O.muricata*, *O.ochrantha*, *O.glabra*, and *O.latibracteata*, showed smooth and undulating inner margins of the outer stomatal ledge (Figs 5, 7, 9), while *O.bicolor* showed only a smooth inner margin of the outer stomatal ledge (Figs 5, 6). In contrast, the remaining species had an undulate inner margin of the outer stomatal ledge. Ornamentation of the outer stomatal ledge was smooth or granular in most species under this study. *O.coerulea* was a notable exception with a banded sediment ornamentation of the outer stomatal ledge (Fig. 7).

### ﻿Trichome characters on the epidermis

Most of the species observed showed trichomes, except for *O.squammulosa* (Table 4; Fig. 9). Two trichome shapes were identified in this genus. Strip-like trichomes, that were present only in *O.ciliata* (Table 4; Fig. 5), and cylindrical trichomes, that were present in all other species (Table 4). Trichrome ornamentation of *O.ochrantha*, *O.ciliata*, and *O.myriophylla* was striate (Table 4; Figs 5, 6), while the remaining species were striate and granular (Table 4).

**Table 4.** Characteristics of the leaf epidermis of *Oxytropis* under scanning electron microscopy.

Species	Adaxial epidermis					Abaxial epidermis				
	Shape of trichromes	Ornamentation of trichromes	Inner margin of outer stomatal ledge	Ornamentation of outer stomatal ledge	Waxy layer of epidermal cells	Shape of trichromes	Ornamentation of trichromes	Inner margin of outer stomatal ledge	Ornamentation of outer stomatal ledge	Waxy layer of epidermal cells
*O.ciliata*	strip-like	striation	undulate	smooth	smooth	absent	absent	smooth	smooth	smooth
*O.muricata*	cylindrical	striation with granular	smooth	smooth	smooth	cylindrical	striation with granular	undulate	granular	granular
*O.falcata*	cylindrical	striation with granular	undulate	granular	scale-like	cylindrical	striation with granular	undulate	granular	granular
*O.ochrantha*	cylindrical	striation	undulate	smooth	granular	cylindrical	striation	smooth	smooth	smooth
*O.bicolor*	cylindrical	striation with granular	smooth	smooth	smooth	cylindrical	striation with granular	smooth	smooth	smooth
*O.racemosa*	cylindrical	striation with granular	undulate	granular	scale-like	cylindrical	striation with granular	undulate	granular	granular
*O.myriophylla*	cylindrical	striation	undulate	granular	smooth	cylindrical	striation	undulate	granular	scale-like
*O.aciphylla*	cylindrical	striation with granular	undulate	scale-like	scale-like	cylindrical	striation with granular	undulate	granular	granular
*O.imbricata*	cylindrical	striation with granular	undulate	granular	granular	cylindrical	striation with granular	undulate	smooth	smooth
*O.coerulea*	cylindrical	striation with granular	undulate	banded sediment	granular	absent	absent	undulate	banded sediment	granular
*O.xinglongshanica*	cylindrical	striation with granular	undulate	scale-like	scale-like	cylindrical	striation with granular	undulate	scale-like	scale-like
*O.glabra*	cylindrical	striation with granular	smooth	smooth	smooth	cylindrical	striation with granular	undulate	granular	granular
*O.kansuensis*	cylindrical	striation with granular	undulate	granular	granular	cylindrical	striation with granular	undulate	granular	granular
*O.melanocalyx*	cylindrical	striation with granular	undulate	scale-like	scale-like	cylindrical	striation with granular	undulate	smooth	smooth
*O.taochensis*	cylindrical	striation with granular	undulate	scale-like	scale-like	cylindrical	striation with granular	undulate	granular	granular
*O.ochrocephala* (XLS)	cylindrical	striation with granular	undulate	scale-like	scale-like	cylindrical	striation with granular	undulate	smooth	smooth
*O.ochrocephala* (HZ)	cylindrical	striation with granular	undulate	scale-like	scale-like	cylindrical	striation with granular	undulate	smooth	smooth
*O.latibracteata*	cylindrical	striation with granular	smooth	smooth	smooth	cylindrical	striation with granular	undulate	smooth	smooth
*O.squammulosa*	absent	absent	undulate	granular	granular	absent	absent	undulate	granular	granular

Note: XLS (Xinglongshan population); HZ (Hezuo population).

### ﻿Cluster analysis

**Figure 10.**
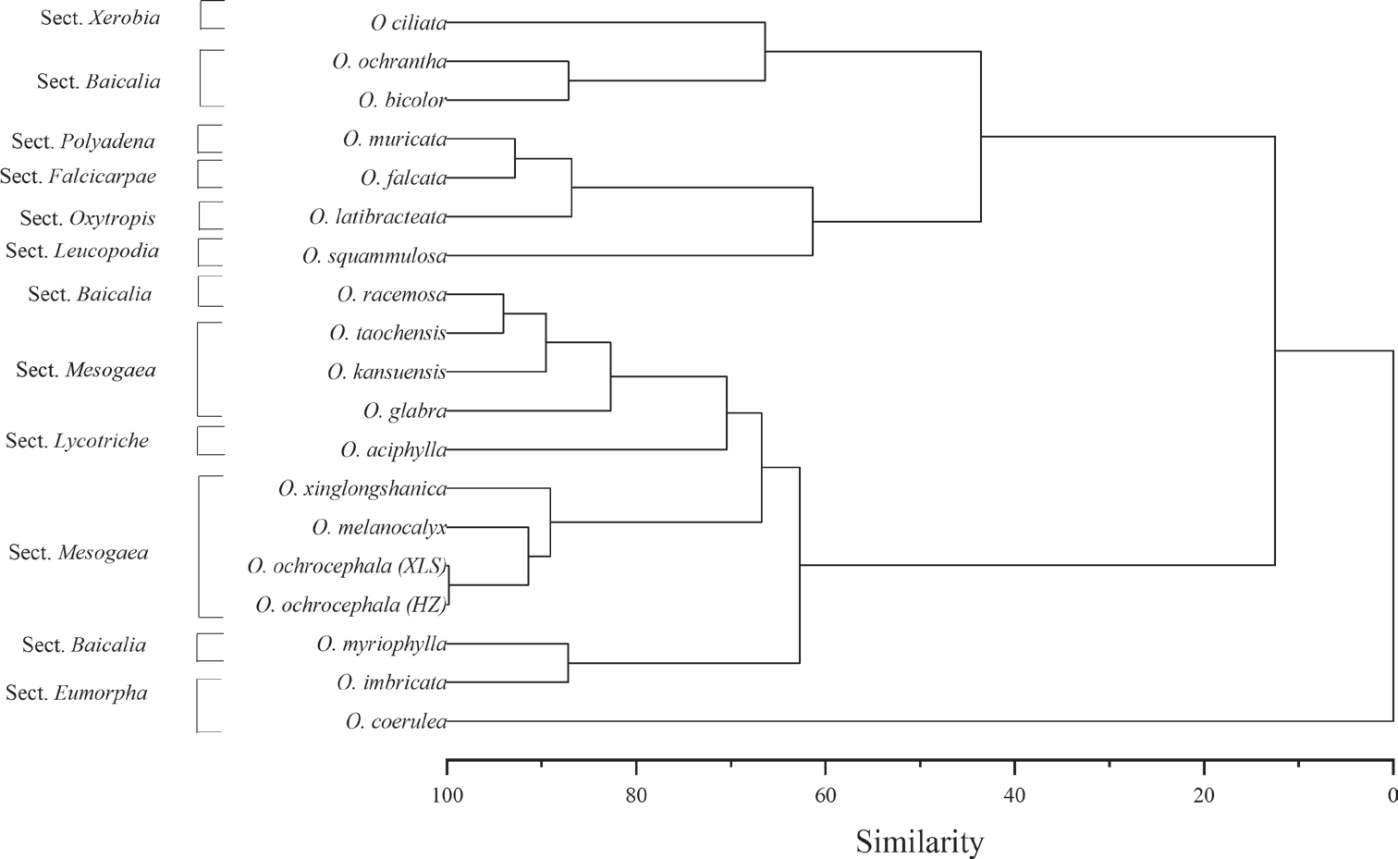
The dendrogram of *Oxytropis* DC. based on the leaf epidermal characteristics.

Cluster analysis reflects the similarity among species based on anatomical characteristics and delimitation of these groups. The phenograms of the quantitative and qualitative data provided four principal clusters (Fig. 10). In the first cluster, *O.ciliata*, *O.ochrantha*, and *O.bicolor* were closely related; in turn, the second cluster included four taxa, *O.falcata*, *O.muricata*, *O.latibracteata*, and *O.squammulosa*. The third cluster comprised *O.racemosa*, *O.glabra*, *O.kansuensis*, *O.aciphylla*, *O.melanocalyx*, *O.taochensis*, *O.ochrocephala* (XLS), *O.ochrocephala* (HZ), *O.xinglongshanica*, *O.myriophylla*, and *O.imbricata*. Lastly, the fourth cluster contained only *O.coerulea*, which was characterized by banded sediments in the outer stomatal ledge.

## ﻿Discussion

Leaf characteristics, such as epidermal micro- and macro-hairs, and stomata, are important for the classification of many genera (Dickison 2000; Yang and Lin 2005; Kadiri and Muellner-Riehl 2021). Previous studies have shown that the anatomical features of the leaf epidermis, such as the shape and anticlinal walls of epidermal cells, are taxonomically significant and can therefore be used for the classification of taxa at the genus or even at the species level (Barthlott et al. 1998; Wissemann 2000; Tomaszewski and Zieliński 2014; Tomaszewski et al. 2019). In this study, there were two main types of leaf epidermal cells: polygonal and irregular; and four different types of pattern of anticlinal walls: straight-arched, sinuolate, undulate, and sinuate. It has been proposed that the pattern of the anticlinal wall may be influenced by habitat; specifically, species in dry environments tend to have a straight arched anticlinal wall, whereas those in humid areas tend to have undulating to sinuous anticlinal walls (Stace 1965; Gifford and Foster 1989). However, in this study, *O.muricata*, *O.falcata*, *O.ochrantha*, *O.bicolor*, and *O.squammulosa* specimens growing in an arid environment exhibited undulate to sinuous anticlinal walls, whereas *O.taochensis* and *O.ochrocephala* specimens found in humid environments exhibited straight arched anticlinal walls. Therefore, our results do not support the aforementioned hypothesis. A similar phenomenon was observed in the study of leaf epidermal traits in Piperales (Song et al. 2020). Furthermore, the shape and anticlinal walls of epidermal cells in *O.ochrocephala* were highly consistent in different populations, indicating that the shape of epidermal cells and the pattern of anticlinal walls were constant within species. Notably, *O.ochrocephala* and *O.kansuensis* are two species easily confused within *Oxytropis*, as they are morphologically difficult to distinguish and they are both abundant in the Qinghai-Tibetan Plateau region (Zhu et al. 2010). However, according to our observations, these two species can be distinguished based on their wall pattern: *O.ochrocephala* has a sinuate anticlinal wall pattern, whereas *O.kansuensis* has an undulating wall pattern. Thus, anticlinal wall pattern might be considered as a useful taxonomic marker for some *Oxytropis* species. However, similar epidermal cell shapes and anticlinal wall patterns exist in other species of the genus *Oxytropis* and other groups of Fabaceae (Zou et al. 2008; Ren et al. 2007). Therefore, epidermal cell shape and anticlinal wall patterns need to be considered in combination with other macro-morphological features classifying the species within the genus *Oxytropis*.

Studies on stomata can have great taxonomic significance for the delimitation of different levels of taxa (Kothari and Shah 1975). Carpenter and Smith (1975) showed that variability in stomatal frequency is taxonomically important at the genus level, whereas Carlquist (1961) emphasized the contribution of stomatal size variation to delimiting species within a genus. In *Oxytropis*, a wide range of variability was observed for stomatal quantitative parameters, such as stomatal density, size, and index. Our results indicated that the quantitative stomatal traits have limited taxonomic value, as they are strongly influenced by environmental factors, such as CO_2_ levels and light intensity (Metcalfe and Chalk 1950; Royer 2001; Rossatto and Kolb 2010). However, stomatal distribution and types are considered an important taxonomic criterion for taxonomic value, especially at higher taxa (Metcalfe and Chalk 1950; Patil and Patil 1987). Thus, for example, we found that the anomocytic stomata type is a common feature in *Oxytropis* that may be used to elaborate the phylogenetic relationships among genera, in combination with stomatal data from other genera. These findings support the concept that genus *Oxytropis* is a monophyletic group (Zhu and Ohashi 2000).

Further, trichomes and their characteristics provide important information for plant identification. The type of indumentum and its presence or absence may serve as diagnostic features for species or genus recognition, as has been recognized in some groups such as Asteraceae (Adedeji and Jewoola 2008; Krak and Mráz 2008), Brassicaceae (Beilstein et al. 2006), Fabaceae (Chukwuma et al. 2014), and Lamiaceae (Eiji and Salmaki 2016). In addition, large plant taxa often share a common pattern of trichome structure. For example, chandelier-shaped trichomes with branches of whorls are characteristic of Platanaceae (Carpenter et al. 2005); peltate or scale-like hairs are typical of Eleagnaceae (Mishra 2009), and three-celled uniseriate hairs are common in Proteaceae (Johnson and Briggs 1975). In the genus *Oxytropis*, the trichome type of the investigated species was simple hair. This is consistent with the results of previous studies on *Oxytropis* (Karaman et al. 2009; Lu 2011). Furthermore, we found that *O.ciliata*, belonging to Section Xerobia (Zhu et al. 2010), has strip-like trichomes (margin ciliates) that distinguish this species from other species in this study. The trichrome ornamentation of most *Oxytropis* species was consistent, indicating that trichrome ornamentation appears to be of a low taxonomic value for distinguishing sections and species. However, owing to sample size limitations, the systematic significance of *Oxytropis* trichomes needs to be based on a more comprehensive sampling.

In this study, six species, including *O.glabra*, *O.kansuensis*, *O.melanocalyx*, *O.taochensis*, *O.ochrocephala*, and *O.xinglongshanica*, all belonging to section Mesogaea, clustered together. Our results of cluster analysis are largely consistent with that of the classification of species and sections based on macro morphological data (Zhu et al. 2010), indicating that leaf epidermal micro characteristics might be valuable in understanding systematics of genera at the section level. Bunge (1874) established the section Gobicola in 1874, which contained only *O.racemosa*. This treatment was recognized by the FRPS, but section Gobicola was merged into section Baicalia in Flora IntraMongolica and FOC (Fu 1989; Zhang 1998; Zhu et al. 2010). However, our results do not support the interpretation of Flora IntraMongolica and FOC. In this study, *O.racemosa* and some species of the section Mesogaea, such as *O.glabra* and *O.kansuensis*, clustered together into one clade, indicating that the systematic position of *O.racemosa* needs to be reconsidered (Fig. 10). In addition, based on the results of quantitative taxonomy, Wang (2005) advocated that section Leucopodia, which only contains *O.squammulosa*, should be merged with section Xerobia. Our results clearly do not support this treatment, because *O.squammulosa* did not cluster together with *O.ciliata* in section Xerobia (Fig. 10). Moreover, different populations of *O.ochrocephala* clustered together into one group, which demonstrates that leaf epidermal traits are useful for the identification of taxa at the species level. Therefore, foliar epidermis traits of *Oxytropis* can be used as taxonomic markers for identification at the infrageneric classification level.

There is no comprehensive phylogenetic study on the genus *Oxytropis*. Furthermore, although several studies have applied DNA barcodes such as ITS, trnL-F, and psbA-trnH to explore the molecular phylogeny of *Oxytropis* in Northwestern China, the low genetic divergence of the above barcodes among the species makes it difficult to distinguish species within the genus as well as to resolve phylogenetic relationships between sections (Li et al. 2011; Gao et al. 2013; Lu et al. 2014). Therefore, the reliability of epidermis characters in terms of phylogeny cannot be affirmed. More detailed molecular phylogenetic studies with a broader taxon sampling are required to find correlations between epidermis characteristics and classification of the genus.

## ﻿Conclusions

Our results suggest that leaf epidermis can be used as potential taxonomic markers for infrageneric classification of *Oxytropis*. The shape of epidermal cells and the pattern of the anticlinal wall were constant within species, and are useful for species delimitation in the genus *Oxytropis* when combined with other macroscopic traits. Trichome shapes can be useful characteristics to distinguish *O.ciliata* from other investigated species. Although quantitative stomatal characteristics were not effective diagnostic characteristics because of the considerable variation within the same taxa, it nevertheless plays an important role in cluster analysis. Results of cluster analysis are largely consistent with the classification of species and sections based on macro morphological data, indicating that foliar epidermis characteristics of *Oxytropis* can be used as taxonomic identification markers infrageneric classification level. Lastly, our results support the delineation of the sect. Leucopodia as an independent section, while not supporting the treatment of merging the sect. Gobicola into the sect. Baicalia.
